# Evaluation of Polymerase Chain Reaction (PCR) with Slit Skin Smear Examination (SSS) to Confirm Clinical Diagnosis of Leprosy in Eastern Nepal

**DOI:** 10.1371/journal.pntd.0005220

**Published:** 2016-12-27

**Authors:** Shraddha Siwakoti, Keshav Rai, Narayan Raj Bhattarai, Sudha Agarwal, Basudha Khanal

**Affiliations:** 1 Department of Microbiology, B. P. Koirala Institute of Health Sciences, Dharan, Nepal; 2 Department of Dermatology and STD, B. P. Koirala Institute of Health Sciences, Dharan, Nepal; Fondation Raoul Follereau, FRANCE

## Abstract

**Background:**

Detection of *Mycobacterium leprae* in slit skin smear (SSS) is a gold standard technique for the leprosy diagnosis. Over recent years, molecular diagnosis by using PCR has been increasingly used as an alternative for its diagnosis due to its higher sensitivity. This study was carried out for comparative evaluation of PCR and SSS microscopy in a cohort of new leprosy cases diagnosed in B. P. Koirala Institute of health Sciences, Dharan, Nepal.

**Methodology/Principal Findings:**

In this prospective crossectional study, 50 new clinically diagnosed cases of leprosy were included. DNA was extracted from SSS and PCR was carried out to amplify 129 bp sequence of *M*. *leprae* repetitive element. Sensitivity of SSS and PCR was 18% and 72% respectively. Improvement of 54% case detection by PCR clearly showed its advantage over SSS. Furthermore, PCR could confirm the leprosy diagnosis in 66% of AFB negative cases indicating its superiority over SSS. In the paucibacillary (PB) patients, whose BI was zero; sensitivity of PCR was 44%, whereas it was 78% in the multibacillary patients.

**Conclusions/Significance:**

Our study showed PCR to be more sensitive than SSS microscopy in diagnosing leprosy. Moreover, it explored the characteristic feature of PCR which detected higher level of early stage(PB) cases tested negative by SSS. Being an expensive technique, PCR may not be feasible in all the cases, however, it would be useful in diagnosis of early cases of leprosy as opposed to SSS.

## Author Summary

Although leprosy has been eliminated at the national level, but region wise eastern Nepal has still the higher number of leprosy cases. Early diagnosis and then timely treatment is very crucial to prevent disabilities due to leprosy. Slit-skin smear (SSS), the routine diagnostic method was compared with the molecular method polymerase chain reaction (PCR) for the detection of new clinically diagnosed cases at BPKIHS. PCR was found to be much more superior than SSS in its diagnostic efficacy detecting more than half number of cases missed by SSS. Moreover our finding of PCR in detection of early stages was nearly 50% where SSS are mostly negative on such spectrum. Therefore, this costly but sensitive diagnostic tool PCR was found to be a better option in detection of early cases of leprosy in comparison to SSS.

## Introduction

Leprosy is a chronic infectious disease which mainly affects the skin, nasal mucosa, and peripheral nerve. It is caused by *Mycobacterium leprae*, an acid-fast bacillus which is transmitted via droplets from the nose and mouth during close contacts with untreated cases [1]. It eventually leads to disability, disfiguration and social stigma for the rest of patient’s life if untreated. Therefore, early detection of *M*. *leprae* is the key element to timely identification and treatment of patients before nerve involvement occurs.

Diagnosis of leprosy has traditionally been based on clinical examinations and skin smears [2]. Microscopy has advantage of being easily available at peripheral and referral centers but as its detection limit is 10^4^ bacilli/ml, it suffers from low sensitivity [3]. *M*. *leprae* does not grow in vitro. In contrary to microscopy, DNA based PCR have shown superior performance to detect bacilli in clinical samples [4–8]. However, their application is not wide in resources poor countries.

Elimination of leprosy as a public health problem at the global level was achieved by 2000; elimination was defined as a reduction in prevalence to <1 case per 10,000 population [9]. Nepal has achieved the elimination goal in December 2009, but then the incidence of new case & prevalence rate has increased from 0.77 to 0.79, 0.84, 0.82 and 0.83 respectively during fiscal year (2009/10) to (2013/14)[10]. Despite achieving the official leprosy elimination goal, it is still considered as a smoldering problem as several cases are under reported to health care facilities. It shows that post elimination phase is more challenging as to maintain the low number of case load which is entirely dependent upon the precise diagnostic tools. In this context, present study was conducted to compare the performance of PCR with the existing slit skin smear for detection of clinically diagnosed cases of leprosy.

## Materials and Methods

### Study design and participants

This is a prospective crossectional study. Consecutive new clinically diagnosed cases of leprosy who presented to department of dermatology and venereology during April 2012 to March 2013 were considered for enrollment in the study. Laboratory investigations were carried out in the department of microbiology. Those patients who did not give consent and those patient under treatment or having past history of treatment received were not included.

### Case definition

A case of leprosy was defined by the presence of any one of the following cardinal signs [11].

1. Characteristic skin lesion with partial or total loss of sensation in the affected skin lesion or in the area of the skin supplied by the peripheral nerve involved with or without the presence of thickened nerves.
2. Presence of AFB in skin smears.

Cases were clinically categorized according to Ridley-Jopling classification(RJ)[12] into: Tuberculoid (TT), Borderline tuberculoid (BT), Borderline (BB), Borderline lepromatous (BL) and Lepromatous (LL). Likewise the cases were also classified according to WHO classification [13] system as Paucibacillary (PB) and Multibacillary (MB).

### Ethics statement

Ethical clearance to undertake the study was obtained from Institutional review board of B. P. Koirala Institute of Health Sciences (BPKIHS), Dharan, Nepal. Written informed consent was obtained from all the adult patients.

### Test methods

#### SSS microscopy (Reference test)

Slit-skin smears were processed at the Dermatology department. Skin smears were taken from 6 sites (two earlobes, two from forehead just above the medial aspect of eyebrows, one from lesion and one from apparent normal skin) from a patient. A moderate thick smear of about 5mm diameter was made. Smears were air dried and submitted to microbiology laboratory for microscopy. All the slit-skin smears were then stained for routine diagnosis following the modified AFB staining technique [14]. SSS were interpreted as positive if *M*. *leprae* bacteria were seen and negative if no bacteria were seen examining the entire smear. Bacteriological index (BI) was graded of the positive SSS.

SSS was chosen as the reference test because despite its low sensitivity (10–50%, depending upon expertise of laboratory workers), SSS remains gold standard for all diagnostic techniques due to specificity of nearly 100% [15].

#### DNA extraction from SSS

One lesional site of SSS per patient was considered for PCR. Slides were treated with xylene for 2min to remove the oil and washed with water. After complete drying, Phosphate-buffered saline(pH 7.4, 1x PBS: 10 mM PO_4_^3−^, 137 mM NaCl, and 2.7 mM KCl) 20 μl was added to smear, then scraped with surgical blade and collected in a 1.5 ml micro centrifuge tube. Lysozyme (2mg/ml; 4 μl: HIMEDIA, Mumbai, India) was added, mixed gently, centrifuged at 10,000 rpm for 5sec and then incubated at 37°C for 1hr. Then 150μl of lysis buffer (1 mg/ml Proteinase K and 0.05% Tween 20) was added. About 30μl of 10% Sodium dodecyl sulfate (Sigma Aldrich, Switzerland) was also added to the lysate. The tube containing lysate was centrifuged 30 sec at 10,000 rpm, then vortex for 10–15 sec and again centrifuged for 30 sec. And then it was incubated at 60°C for overnight, then temperature was set at 94°C and the reaction was terminated at 94°C for 15 minutes [16,17]. Then purification of DNA by extraction with Phenol:Choloroform method was done as per the method described elsewhere [18].

#### PCR amplification assay (Index test)

**Fig 1:**
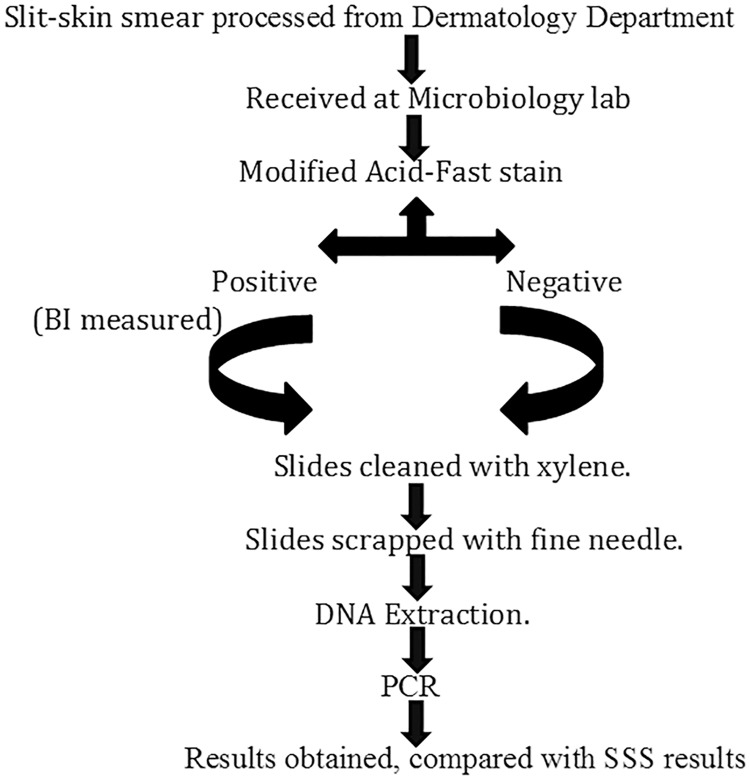
Flow chart of overview of methods.

We used a PCR assay targeting the *M*. *leprae*-specific repetitive element (RLEP). PCR reaction was carried out in 25 μL of PCR reaction mix consisting of 2.5 μL of DNA sample, 200 μM of dNTP master mix (Eurogentec, Belgium), 1X coralloid reaction buffer (Qiagen, Benelux), 1.5 mM MgCl_2_ (Qiagen, Benelux), 1 unit of hot start Taq plus DNA polymerase (Qiagen, Benelux) and 0.4μM of each primer (LP1 and LP2)[19](Macrogen, South Korea). The reaction mixtures were amplified in an Eppendorf Master cycler EP Gradient S with ESP heated block control. Cycling conditions were: (i)1 cycle consisting 95°C for 5 minutes, followed by 58°C for 2min and 72°C for 2min (ii) 45 cycles, each consisting of 30 sec at 94°C, 30 sec at 58°C, and 1min at 72°C and (iii) a final extension step of 10 min at 72°C. The 129-bp amplicon was detected on a 2% agarose gel after Ethidium Bromide staining. PCR was interpreted as positive if 129-bp amplicon was detected and negative if such amplicon not detected. Negative samples were submitted to a new amplification after 1/10 dilution of the template DNA. Positive and negative controls were included in each PCR run. SSS microscopy positive samples were employed as a positive control while DNA free milli-Q water was included as a negative control. The microbiologist performing the PCR was blinded about the clinical and SSS status of patient. A flowchart representing the workflow of steps of the methods is illustrated in Fig 1.

### Sample size calculation and data analysis

Previous study[20] reported sensitivity of PCR (index test) and SSS (reference test) of 86% and 60% respectively. With power of 90% with α error 5% (one sided), fifty patients were required in this study.

Data were statistically described in terms of range, mean ± standard deviation (SD), frequency (number of cases), relative frequency (percentages), and confidence interval when appropriate. The statistical significance of the differences in sensitivities between PCR and SSS microscopy were assessed by means of Chi square test, Fisher’s exact test and kappa test. SPSS (version 15; Chicago, IL) was used for statistical analysis. In addition, the ROC curve was analyzed for PCR diagnosis with RJ Clinical Types by R version 3.0.3 (www.r-project.org) [21] applying the package pROC [22].

## Results

### Participants

**Fig 2:**
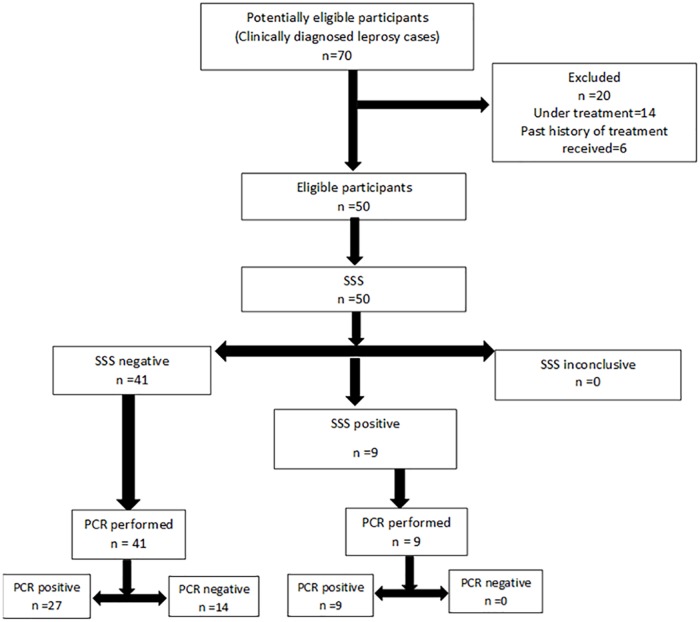
Flow diagram of participants.

The study included 50 patients who met the criteria of case definition of leprosy and did not have history of past treatment for leprosy. Consecutive SSS microscopy was performed from 50 patients within 1 year and then, PCR was performed on all 50 SSS. The flow of participants through the study is shown in Fig 2.

There were 27 (54%) male and 23 (46%) female. Mean age of patient was 42.12 ± 17.11 years (range: 10–75 years; median: 44.50 years). Majority (66%) of the lesions had well defined margin. Nerve involvement was found in 44 (88%) of the patients on peripheral nerve examination with ulnar nerve being commnest. Eye involvement was present in 9(18%).

### Test results

**Fig 3:**
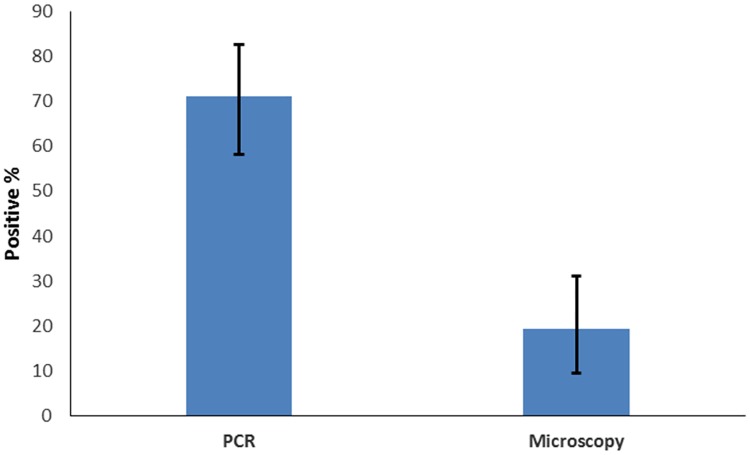
Sensitivity of Microscopy and PCR with Confidence interval (CI).

**Fig 4:**
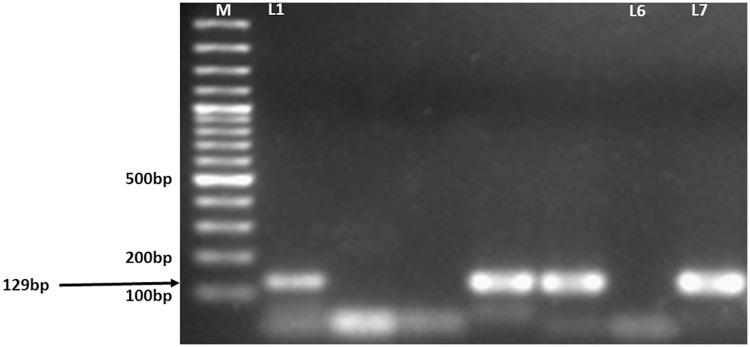
Agarose gel image of the *M*. *leprae* PCR products. M; Molecular markers. Lanes 1–5; Patient samples. L6; Negative control. L7; Positive control.

Out of 50 patients, microscopy was positive in 9 [18%; 95%CI,(9.54–31.02)]cases. In contrast, PCR detected *M*. *leprae* in 36 [72%; 95%CI,(58.24–82.62)]cases (Fig 3). There was a 129 bp DNA band in agarose gel electrophoresis, indicating presence of *M*. *leprae* as shown in Fig 4.

A total of 9(18%) cases tested positive by both diagnostic tools i.e. modified AFB staining and PCR. There was no case in which microscopy was positive and PCR was negative (Table 1). PCR confirmed the diagnosis in 27 (66%) out of 41 skin smears which were AFB negative.

**Table 1:** Correlation of results of PCR with skin-smears. **(n = 50)**.

PCR	SSS(Positive)	SSS(Negative)	Total
Positive	9(18%)	27(54%)	36(72%)
Negative	0(0%)	14(28%)	14(28%)
Total	9(18%)	41(82%)	50(100%)
Kappa	0.157		

Further comparative analysis of positivity of AFB in SSS and PCR to the various subgroups as per RJ clinical types and WHO clinical types are depicted in Tables 2 and 3 respectively.

**Table 2:** Correlation of RJ clinical types, results of skin smear for AFB and PCR.

Spectrum of Leprosy	Number of Cases(n)	AFB present (Microscopy)	PCR positive
TT	1(2%)	0(0%)	0(0%)
BT	24(48%)	0(0%)	12(50%)
BB	1(2%)	1(100%)	1(100%)
BL	14(28%)	2(14%)	14(100%)
LL	9(18%)	6(67%)	9(100%)
PN	1(2%)	0(100%)	0(0%)

Chi-square(x^2^) = 4.268, p = 0.039.TT-tuberculoid; BT-borderline tuberculoid; BB-borderline; BL-borderline lepromatous;LL-lepromatous; PN-pure neuritic.

**Table 3:** Correlation of WHO clinical types, results of skin smears for AFB and PCR.

	MB(n = 41)	PB(n = 9)	Total(n = 50)
PCR positive	32(78%)	4(44%)	36(72%)
AFB present (Microscopy)	9(22%)	0(0%)	9(18%)
Fisher’s exact test	3.243	-	4.268
*p-*value	0.167	-	0.047

**Fig 5:**
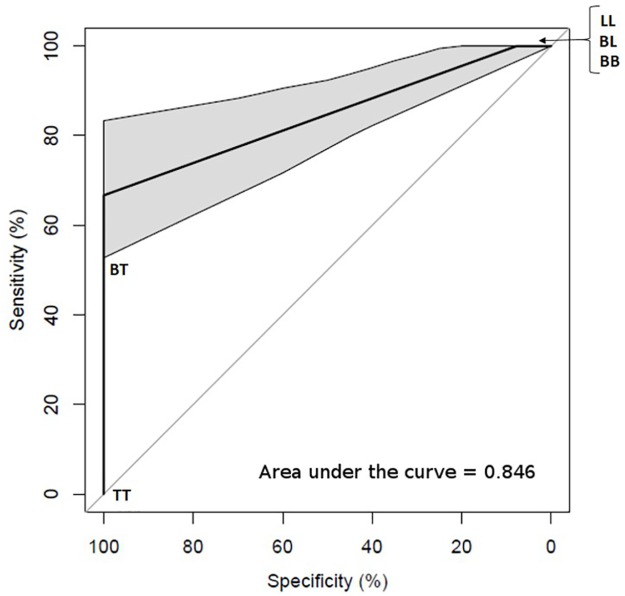
ROC curve of PCR diagnosis with RJ classification.

Receiver Operating Characteristic (ROC) curve analysis was performed for PCR with RJ classification (Fig 5). The diagnostic accuracy was 0.846 (area under curve; AUC = 0.846, 95% CI = 0.7698–0.9225).

## Discussion

SSS being cheap and minimally invasive, are the main tool for diagnosis of leprosy in the developing countries where most cases are newly detected [9]. However, this conventional technique has the disadvantage of being less sensitive [3]. There is need for a more sensitive diagnostic tool for early diagnosis of leprosy cases to prevent the deformities and disabilities. Several studies have reported successes to detect *M*. *leprae* by PCR in SSS showing its clear advantage over SSS microcopy [8,16]. Therefore, the present study was carried on 50 new patients comparing the routine conventional SSS microscopy with PCR on SSS in the leprosy diagnosis. Our study demonstrated a positive yield in 18% of all cases of clinically diagnosed leprosy by SSS microscopy, while 72% were positive by PCR. As the 95% confidence interval of sensitivity of microscopy and PCR do not overlap in Fig 3, therefore, this result showed the difference in the diagnostic efficacy of these two diagnostic tool to be statistically significant [23]. Moreover, the ROC curve of PCR indicates it as a good diagnostic tool since the AUC is 0.846 as shown in Fig 5 [24]. Hence, this study shows the diagnostic efficacy of PCR is more efficient than microscopy in diagnosing leprosy. The low sensitivity of SSS microcopy can be explained by individual observer variation and low load of bacteria in SSS. An improvement of 54% in the case detection rate as compared to the results of SSS alone definitely mirrors out the better diagnostic efficiency of PCR over microscopy. Furthermore, the PCR sensitivity could have been greater, but xylene and other chemical components used during DNA extraction may have inhibited the PCR. Our findings of PCR with overall 72% yield was comparable to that of Kamal et al [25] with 72% overall yield with *in situ* PCR on SSS. However, this rate was still lower as compared to the studies conducted in Thailand [8] and Kolkata [20] in which PCR positivity was noted to be 87% and 82.3% respectively in the biopsy samples. This difference can be explained by the low level of DNA in SSS due to less amount of tissue as compared to biopsy. It is interesting to note that our results were still better with 12% more than the Dayal et al [26], study in which *in situ* PCR was used in skin biopsy resulting into positivity of 60%. Difference in the nature of the PCR primer (36kDa antigen gene) as compared to that of ours (RLEP LP1/LP2) may be the possible reason for the discrepancy in the findings. Compared with 36-kDa antigen gene primers, the RLEP primers were found to be 1000-fold sensitive in detection of *M*. *leprae* DNA in a study performed in London [19].

In the present study, 18% of the total cases were SSS positive with 100% in BB, 67% in LL and 14% in BL subgroups. Several studies have reported similar rate sensitivities. In the study by Kamal et al [25], 20% cases were skin smear positive with 44% positivity in BL/LL variety. Similar rate of positivity of SSS was observed by Dayal et al [26] with only 10% positivity out of total cases and that of BL variety. All the PB (BT and TT) cases in the present study tested negative in the SSS, a finding corresponding the results reported by both of the above cited studies in which skin smears were negative in the indeterminate and BT variety. The scarcity of AFB in skin tissues in the early stages (PB) usually causes difficulty in microscopic diagnosis. Our study found the PCR positivity rate of 66% in AFB negative cases which was similar to other studies findings of 65% [20] and 72.7% [26] in the cases which were AFB negative. This highlights the superiority of PCR over SSS which could confirm the leprosy diagnosis in more than half of the cases missed by SSS. Furthermore, our study found the sensitivity of PCR on AFB- positive cases to be 100% indicating no false positive on SSS findings. The number of PCR-positive cases was detected to be higher in MB than in PB patients in our study. As many as 78% of the MB cases were PCR positive, while this rate was only 44% in the PB cases. This result was expected as MB leprosy has a higher bacterial load than PB leprosy. Further, our results are better than the results of another study conducted at Thailand [8] using PCR on SSS. In the latter study, 41.9% positivity was observed in the MB cases and 18.2% in PB cases. But in the study conducted in Vietnam [27], 100% PCR positivity was recorded on the SSS samples from the MB patients, a finding superior to ours. Alternatively, while the results of our PCR test sensitivity was convincing, but this assay was applied only on SSS and not on other clinical specimens which is the limitation of this study.

In summary, our study clearly reveals that PCR is more sensitive than conventional SSS microscopy in diagnosing leprosy. Moreover, this study explored the characteristic feature of PCR which detected higher level of early stage (PB) cases tested negative by SSS. Being an expensive technique, PCR may not be feasible in all the cases, however, it would be useful in diagnosis of early cases of leprosy as opposed to SSS. Besides, PCR is only accessible in few teaching hospitals and referral centers in resource poor country like Nepal. Therefore, early and clinically indeterminate cases can be referred to the referral center for PCR to increase the case detection of leprosy.

## Supporting Information

S1 Checklist STARD Checklist. (DOCX)
